# Interpreting Alcohol‐CV Associations in AF Requires Scrutiny of Drinking Behaviors and Socioeconomic Context

**DOI:** 10.1111/anec.70106

**Published:** 2025-08-01

**Authors:** Yuren Cao

**Affiliations:** ^1^ The Second School of Clinical Medicine Zhejiang Chinese Medical University Hangzhou China

Editors,

We read with great interest the article by Oraii and colleagues (Oraii et al. 2025). Utilizing a large international cohort (the RE‐LY AF registry), the study provides novel insights into the association between different levels of alcohol consumption and cardiovascular outcomes (stroke/systemic embolism, heart failure [HF] hospitalization, major bleeding) in patients with atrial fibrillation (AF). However, when interpreting these important findings, we believe there are noteworthy methodological limitations that warrant attention and improvement in future research.

The study's simplification of alcohol intake to weekly averages, without distinguishing drinking patterns or beverage types, may substantially impact the reliability of its conclusions. Of particular concern is that the concealed risk of binge drinking (≥ 5 drinks per occasion) is obscured by the weekly average grouping. Robust evidence indicates that binge drinking can acutely elevate blood pressure, trigger AF episodes, and promote platelet aggregation, independently increasing stroke risk by approximately 35% (Pooled RR = 1.35) (O'Donnell et al. 2010; Degerud et al. 2021). The “heavy drinker” group (≥ 14 drinks/week) in this study likely included a significant proportion of such high‐risk individuals engaging in binge patterns, yet showed only a non‐significant reduction in stroke risk (aOR = 0.79). Isolating a binge drinking subgroup might reveal significantly elevated stroke and bleeding risks, especially in the context of anticoagulant therapy, potentially reversing the neutral conclusion that alcohol does not increase thrombotic risk. Concurrently, the confounding effect of beverage type was uncontrolled. The potential cardioprotective effects of polyphenols in wine might dilute the overall observed risk, while a predominance of spirits could amplify harm—this heterogeneity introduces bias into the interpretation of dose–response relationships (Castaldo et al. 2019). While this limitation has a lesser impact on the conclusion regarding HF protection (as chronic benefits may align more with regular, moderate consumption), it likely leads to a systematic underestimation of stroke and bleeding risks, diminishing the study's value for clinical decision‐making. Future research urgently needs to integrate dimensions of drinking pattern and beverage type into alcohol categorization; failure to do so risks misleading safety advice for high‐risk populations like binge drinkers.

Stratifying the alcohol‐heart failure association solely by country income, without adjusting for socioeconomic status (SES), healthcare access, or lifestyle factors, fundamentally weakens the conclusion (Allen et al. 2018). The apparent “protective effect” in high‐income countries (aOR = 0.51) likely reflects superior healthcare (e.g., early intervention) and healthier behaviors in high‐SES populations, not alcohol itself. Conversely, elevated HF risk in low‐income drinkers (aOR = 2.18) probably stems from medical resource scarcity (e.g., unavailable diuretics), not alcohol causation. Unmeasured SES confounders like malnutrition further distort results. Since SES adjustment typically halves alcohol's purported cardiovascular benefits, controlling for education/insurance/diet would likely nullify the reported HF risk reduction—potentially unmasking alcohol's true hazards in vulnerable settings. This macro‐level oversimplification conflates health inequities with biological effects.

Nevertheless, Oraii et al.'s work provides valuable insights into global AF patient alcohol patterns. The stark regional heterogeneity in heart failure risk highlights critical research directions. Future studies should incorporate granular drinking patterns (e.g., binge vs. regular), beverage types, and individual socioeconomic/clinical factors. Such integrated analysis will clarify alcohol's true role in AF outcomes and enable personalized recommendations.

## Conflicts of Interest

Th author declares no conflicts of interest.

## Data Availability

Data sharing not applicable to this article as no datasets were generated or analysed during the current study.
